# Cell Surface Processing of CD109 by Meprin β Leads to the Release of Soluble Fragments and Reduced Expression on Extracellular Vesicles

**DOI:** 10.3389/fcell.2021.622390

**Published:** 2021-03-02

**Authors:** Wiebke Lückstädt, Simon Bub, Tomas Koudelka, Egor Pavlenko, Florian Peters, Prasath Somasundaram, Christoph Becker-Pauly, Ralph Lucius, Friederike Zunke, Philipp Arnold

**Affiliations:** ^1^Anatomical Institute, Christian-Albrechts-University Kiel, Kiel, Germany; ^2^Department of Molecular Neurology, University Hospital Erlangen, Erlangen, Germany; ^3^Systematic Proteomics and Bioanalytics, Institute for Experimental Medicine, Christian-Albrechts-University Kiel, Kiel, Germany; ^4^Center for Molecular Medicine Cologne, University of Cologne, Cologne, Germany; ^5^Lab for Retinal Cell Biology, Department of Ophthalmology, University of Zurich, Schlieren, Switzerland; ^6^Biochemical Institute, Christian-Albrechts-University Kiel, Kiel, Germany

**Keywords:** CD109, macroglobulin, meprin β, exosomes, extracellular vesicles, TEM, TEP1r

## Abstract

Cluster of differentiation 109 (CD109) is a glycosylphosphatidylinositol (GPI)-anchored protein expressed on primitive hematopoietic stem cells, activated platelets, CD4^+^ and CD8^+^ T cells, and keratinocytes. In recent years, CD109 was also associated with different tumor entities and identified as a possible future diagnostic marker linked to reduced patient survival. Also, different cell signaling pathways were proposed as targets for CD109 interference including the TGFβ, JAK-STAT3, YAP/TAZ, and EGFR/AKT/mTOR pathways. Here, we identify the metalloproteinase meprin β to cleave CD109 at the cell surface and thereby induce the release of cleavage fragments of different size. Major cleavage was identified within the bait region of CD109 residing in the middle of the protein. To identify the structural localization of the bait region, homology modeling and single-particle analysis were applied, resulting in a molecular model of membrane-associated CD109, which allows for the localization of the newly identified cleavage sites for meprin β and the previously published cleavage sites for the metalloproteinase bone morphogenetic protein-1 (BMP-1). Full-length CD109 localized on extracellular vesicles (EVs) was also identified as a release mechanism, and we can show that proteolytic cleavage of CD109 at the cell surface reduces the amount of CD109 sorted to EVs. In summary, we identified meprin β as the first membrane-bound protease to cleave CD109 within the bait region, provide a first structural model for CD109, and show that cell surface proteolysis correlates negatively with CD109 released on EVs.

## Introduction

**FIGURE 1:**
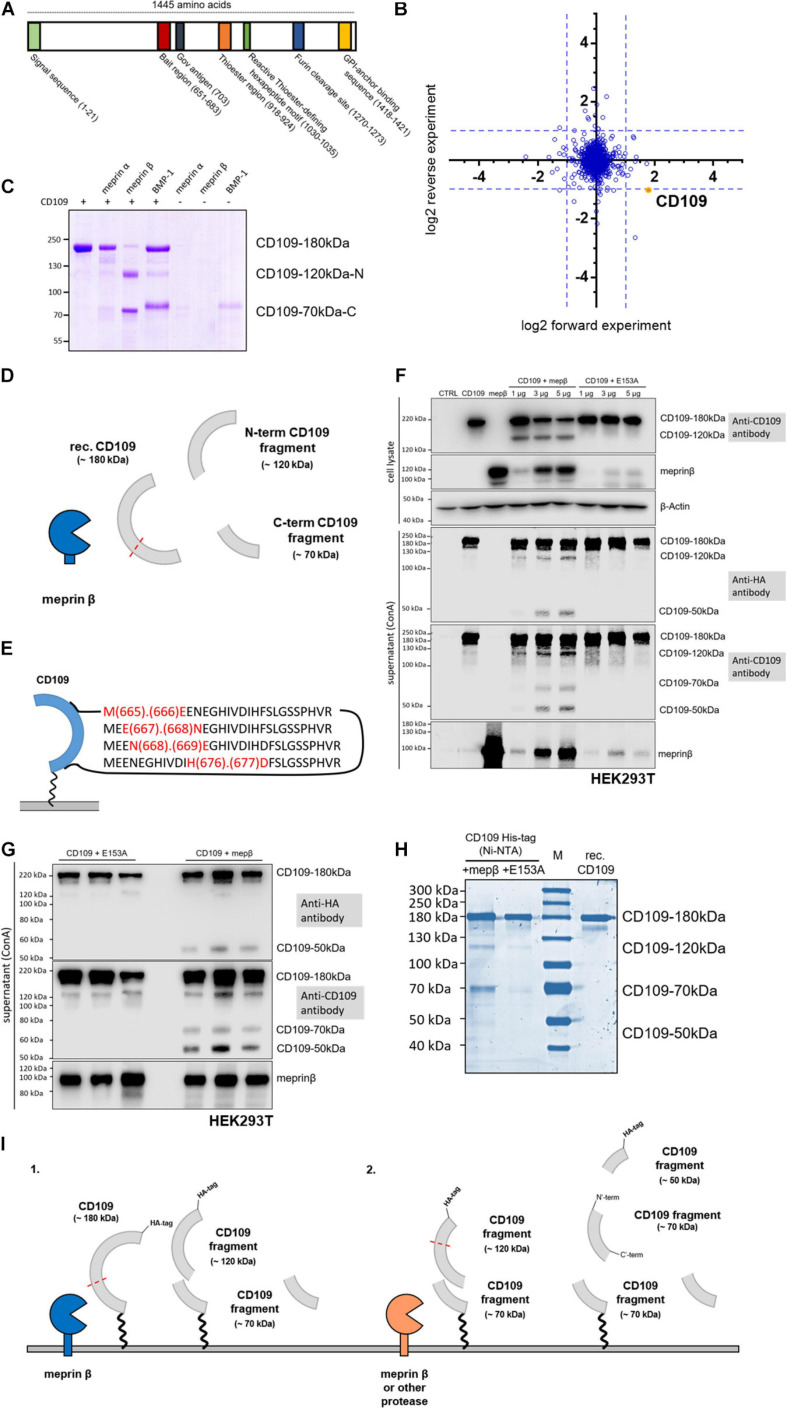
CD109 cleavage by meprin β. **(A)** Schematic structure of CD109 protein with functional regions. In panel **(B)**, proteomic data reflect global protein changes after recombinant meprin β treatment. **(C)** For *in vitro* experiments, recombinant CD109 (3 μg) was incubated with recombinant meprin β, meprin α, or BMP-1 (4 h for meprin β and meprin α, 8 h for BMP-1; 5 nM protease concentration for meprin β and meprin α, 50 nM for BMP-1) and analyzed on a 7.5% Coomassie gel. N-terminal 120-kDa and C-terminal 70-kDa fragment are presented graphically in panel **(D)** and four major cleavage sites were identified by mass spectrometry within the bait region **(E)**. Transient transfections in HEK293T wild-type cells were performed and a representative Western blot in panel **(F)** shows the comparison of single plasmid transfections and double transfections of CD109 N-term HA-tagged and active meprin β (in different concentrations: 1, 3, and 5 μg). In addition, comparable transfections with the inactive meprin β variant E153A as control reflect the various cleavage fragments in cell lysate and supernatant after purification using Concanavalin A (ConA) precipitation. CD109 signals were incubated and detected with anti-HA-tag and anti-CD109 antibody respectively without stripping in between, anti-meprin β antibody, and for cell lysate control anti-β-actin antibody. **(G)** To reduce the effects of double transfections, experiments were done again three times with 5 μg of mepβ and E153A transient transfection into stably transfected CD109 N-term HA-tagged HEK293T cells. **(H)** Full-length CD109 protein and fragments were also shown in Coomassie gel after Ni-NTA purification using cell medium of stably transfected CD109 N-term His-tagged HEK293T cells transiently transfected with active and inactive meprin β (E153A) in comparison with purchased recombinant (rec.) CD109 protein control without protease. **(I)** Possible cleavage events in CD109 by different proteases and the corresponding proteolytic fragments have been outlined.

Cluster of differentiation 109 (CD109) is expressed on primitive hematopoietic stem cells, activated platelets, CD4^+^ and CD8^+^ T cells, and keratinocytes (Lin et al., 2002). CD109 is gaining attention as a potential biomarker as it is found highly expressed in several tumors such as squamous cell carcinoma (Hashimoto et al., 2004; Zhang et al., 2005; Hagiwara et al., 2008; Mo et al., 2020), lung cancer (Hashimoto et al., 2004; Sato et al., 2007; Chuang et al., 2017; Taki et al., 2020), pancreatic cancer (Haun et al., 2014; Moffitt et al., 2015; Arias-Pinilla et al., 2018; Hatsuzawa et al., 2020), breast cancer (Tao et al., 2014), glioma (Shiraki et al., 2017; Minata et al., 2019), and many more (Hagikura et al., 2010; Emori et al., 2013, 2015; Yokoyama et al., 2017). It has been shown that high expression of CD109 in some of those tumors correlates with a bad outcome for patients (Tao et al., 2014; Emori et al., 2015; Chuang et al., 2017; Yokoyama et al., 2017). Many signaling pathways have been proposed to be regulated by CD109 such as inhibition of TGFβ, shown in decreased SMAD2/3 phosphorylation (Finnson et al., 2006; Hagiwara et al., 2008) and TGFβ internalization (Bizet et al., 2011), impact on the activation of the JAK-STAT3 axis (Chuang et al., 2017), or influence on YAP/TAZ signaling (Minata et al., 2019), and more recently EGFR/AKT/mTOR (Lee et al., 2020) as well as EGFR-mediated STAT3 phosphorylation (Mo et al., 2020). CD109 itself is a membrane-bound protein with a glycosylphosphatidylinositol (GPI) anchor first mentioned in Sutherland et al. (1991). Because of its distinct thioester motif, it was classified to belong to the α2 macroglobulin/C3, C4, and C5 family (Lin et al., 2002). Solomon et al. suggest an independent branch of the α2 macroglobulin/complement (AMCOM) gene family, because of its unique organizational properties like a C-term furin cleavage site instead of N-terminal localization as found in other members (Lambris et al., 1993; Solomon et al., 2004) and a GPI anchor (Solomon et al., 2004). The CD109 structure itself has not been solved experimentally yet, but Janssen et al. was able to generate a crystal structure of human complement component C3 and from this deduced a model for domain formation of other members of the α2 macroglobulin family including CD109. It proposed different compositions of the C-terminus between members (Janssen et al., 2005). Proteolytic processing by furin on the secretory pathway was suggested, which should result in full-length cell surface attached CD109 (205 kDa), a 25-kDa fragment also on the cell surface and a 180-kDa fragment detected as soluble CD109 in the cell culture supernatant (Hagiwara et al., 2010). Furthermore, CD109 contains an intact thioester bond (Lin et al., 2002) with a thioester signature sequence (aa 918–924) and a corresponding thioester reactivity defining hexapeptide (aa 1030–1035), as well as a bait region (aa 651–683) (Finnson et al., 2006), which has been shown to be an active target for protease cleavage by the metalloprotease bone morphogenetic protein-1 (BMP-1) (schematic overview, Figure 1A) (Delolme et al., 2015). Hockla et al. showed that mesotrypsin sheds CD109 from the cell surface of a breast cancer cell line (HMT-3522 T4-2) resulting in enhanced malignant growth (Hockla et al., 2010). Proteases can function as a signaling switch by cleaving membrane proteins and induce signal transduction within the same cell (cis) and/or across cell boundaries (trans) (Lokau et al., 2017; Zunke and Rose-John, 2017). Here, we focus on the interaction of CD109 and meprin β, a zinc-dependent multidomain metalloprotease of the astacin family (Bond and Beynon, 1995). Meprin β is found on the cell surface of proximal tubule cells of the kidney, enterocytes of the small intestine and colon, as well as endothelial cells in blood vessels, in keratinocytes of the epidermis, and in leukocytes (Prox et al., 2015; Arnold et al., 2017). It was also shown that meprin β can promote transendothelial cell migration by ectodomain shedding of CD99 (Bedau et al., 2017a), and hence might play a role in cancer metastasis as proposed for CD109 as well. Meprin β is also expressed in mesenteric lymph nodes, in which it is affecting the intestinal leukocyte migration (Crisman et al., 2004). Furthermore, the isoform meprin β′ mRNA, containing an additional 5′ sequence, was discovered in human colon adenocarcinoma cell line (HT29-18C1) (Dietrich et al., 1996), human breast cancer cell lines (MCF-7 and SK-BR-3), human osteosarcoma cell line (U-2 OS), and the human pancreatic cancer cell line (BxPC-3), indicating its aspect in cancer (Matters and Bond, 1999). We could also show that single amino acid exchange variants of meprin β identified in cancer patients alter its proteolytic activity on the cell surface (Schäffler et al., 2019). In addition to direct cell–cell interaction, communication between cells can also occur through extracellular vesicles (EVs), which carry proteins like CD109 (Sakakura et al., 2016; Yamamoto et al., 2019). In recent years, EVs emerged as important players during metastasis (Hood et al., 2011; Peinado et al., 2012) as well as promotion of angiogenesis (Hood et al., 2011; Kuriyama et al., 2020), and since there have been reports on high CD109 levels in tumors worsening patient outcome as described above, the role of CD109-carrying EVs and their impact on tumors and metastases need further investigation and, to date, remain mostly unclear.

In this study, we focus on proteolysis of CD109 at the cell surface as a regulatory event. We identify cleavage sites utilized by the metalloprotease meprin β and show that proteolytic cleavage reduces secretion of CD109 via EVs. We also expressed and purified a soluble version of CD109 and used single-particle analysis to produce a low-resolution (15 Å) 3D reconstruction of CD109, which, together with molecular modeling, results in an experimentally derived structural model of CD109, indicating its position at the cell surface.

## Materials and Methods

Unless described otherwise, chemicals and detergents were purchased from Carl Roth GmbH & Co. KG, Karlsruhe, Germany, and disposable material were obtained from SARSTEDT AG & Co. KG, Nümbrecht, Germany.

### Cell Lines, Cultivation, and Transient Transfection

HEK293T cells were purchased from Leibniz Institute DSMZ-German Collection of Microorganisms and Cell Cultures GmbH (Braunschweig, Germany) and cultivated in Dulbecco’s Modified Eagle’s Medium high-glucose medium (DMEM, Gibco^TM^, Carlsbad, CA, United States) with 10% fetal bovine serum (FBS, PANBiotech, Aidenbach, Germany) and 1% Penicillin/Streptomycin (Applied Biological Materials Inc., Richmond, Canada) at 37°C, with 5% CO_2_, and 95% humidity. Transient transfection was performed as described before (Arnold et al., 2015). Briefly, cells were grown to 60–70% confluence, and medium was changed before the addition of the transient transfection mixture. This contained (for a 10-cm dish) 5 μg of DNA plasmid and 15 μl of polyethylenimine (PEI, 1 μg/ml, Merck Millipore, Billerica, MA, United States) in 300 μl of DMEM without FBS incubated at 37°C for 30 min. After 16 h, medium was changed again to DMEM without FBS for the next 24 h to remove PEI and to produce good working conditions for meprin β.

HEK293F cells (Thermo Fisher Scientific Inc., Waltham, MA, United States) were cultured in serum- and protein-free FreeStyle 293 expression medium without additional supplements (Thermo Fisher Scientific Inc., Waltham, MA, United States) at 37°C, with 8% CO_2_, 95% humidity, and agitation at 125 rpm. Prior to transfection, the cell count was determined by trypan blue staining (Merck Millipore, Billerica, MA, United States) and Cellometer^®^ Auto T4 Plus (Nexcelom Bioscience, Lawrence, MA, United States). The cells were seeded at a density of 1 × 10^6^ cells/ml in fresh media and incubated for 30 min. Subsequently, they were transfected again with three parts PEI to one part DNA diluted in Opti-MEM I Reduced Serum Media (Thermo Fisher Scientific Inc., Waltham, MA, United States). For increased overexpression, cells were supplemented with 3.5 mM valproic acid (Merck Millipore, Billerica, MA, United States) 12 h post transfection. It has been determined that media harvest after 96 h produces the highest yield.

### Generating Stable CD109 HEK293T Cell Lines

Both expression constructs pCMV3-SP-CD109-N-His (Sino Biological Inc., Peking, China) and pCMV3-SP-CD109-N-HA (Sino Biological Inc., Peking, China) contain an antibiotic resistance in mammalian cell systems against hygromycin B. To determine the minimum antibiotic concentration needed to kill all the non-transfected cells in 1 week, the amount of hygromycin B was determined by performing a kill curve. Afterwards, the concentration of 0.15 mg/ml and 0.2 mg/ml hygromycin B was chosen. HEK293T cells (passage no. 6) were transfected as described above and changed after 16 h onto selective (hygromycin B-containing) medium with 10% FBS. Every other day, medium was discarded and cells were washed with Dulbecco’s phosphate-buffered saline (DPBS, GibcoTM, Carlsbad, CA, United States) and given fresh selective medium over 9 days. Polyclonal colonies were resuspended in selective medium and seeded statistically 1 cell/well in a 96-well plate, again for 9 days. Monoclonal cell colonies were then transferred to grow in 6-well plates, checked for protein expression via Western blot of supernatant and cell lysates, and used for experiments. Stable cell lines HEK293T CD109 N-term HA-tag and N-term His-tag between passages 8 and 12 (after transfection) have been used for studies.

### Cell Lysis, Concanavalin A Precipitation, Coomassie Blue Staining, and Western Blot

Cell lysis, Coomassie Brilliant Blue staining, and Western blotting were performed as described previously (Arnold et al., 2015). Shortly, for preparing cell lysates, cells were harvested and transferred onto ice. After washing the cells three times with ice-cold DPBS, centrifuging in between for 3 min at 2,000 × *g*, 4°C, lysis buffer [1% Triton X-100, Complete protease inhibitor cocktail (Roche, Penzberg, Germany) in DPBS] was added and the suspension was incubated for 30 min on ice. Afterward, cell lysates were centrifuged at 13,000 × *g* for 15 min at 4°C to remove cell debris. Protein amount was determined using Pierce^TM^ BCA Protein Assay Kit (Thermo Fisher Scientific Inc., Waltham, MA, United States). Supernatant was then mixed with sodium dodecyl sulfate (SDS)-containing loading buffer and proteins were denatured for 10 min at 98°C. After separation by SDS-PAGE, proteins were either stained with Coomassie Brilliant Blue R-250 or transferred to a nitrocellulose 0.2-μm (GE Healthcare, Boston, MA, United States) membrane, blocked with 3% milk or BSA (bovine serum albumin) in Tris-buffered saline (TBS; 100 mM Tris–HCl and 685 mM NaCl, pH 7.5) for 1.5–2 h and then incubated with the primary antibody overnight at 4°C (see Table 1). Afterward, the membranes were washed with TBS-T (TBS, 0.25% Tween 20, and 1% Triton X-100, pH 7.5), and the appropriate secondary antibody was added for 1 h. The membranes were washed again with TBS-T and were then developed using Vilber Lourmat Peqlab FUSION SL Gel Chemiluminescence Documentation System (PEQLAB Biotechnologie GmbH, Erlangen, Germany) or Amersham^TM^ Typhoon^TM^ Biomolecular Imager infrared imaging system (for Coomassie gel detection). Concanavalin A (Merck Millipore, Billerica, MA, United States) (ConA) precipitation from cell culture supernatants was performed as described previously (Arnold et al., 2017).

**TABLE 1:** List of primary antibodies.

**Antigen**	**Host species**	**Dilution**	**Retailer**
Anti-β-Actin	Rabbit	1:5000	Merck KGaA, Darmstadt, Germany
Anti-Human CD109 (TEA 2/16)	Mouse	1:1000	BD Biosciences, San Jose, CA, United States
Anti-Meprin β	Rabbit	1:1000	Pineda Antikörper-Service, Berlin, Germany
Anti-CD63	Rabbit	1:1000	System Biosciences, LLC, Palo Alto, CA, United States
HA-tag (6E2)	Mouse	1:1000	Cell Signaling Technology, Inc., Danvers, MA, United States

### Generation of Expression Construct via Site-Directed Mutagenesis

The expression construct pCMV3-SP-CD109-N-His (Sino Biological Inc., Peking, China) was used as a template to generate a mutant construct of the soluble protein. The individual mutagenesis primer was designed as shown: 5′-TTGTGAGGATTGAGCTTCAGGC-3′ and 5′-GCCTGAAGCTCAATCCTCACAA-3′, synthesized by Thermo Fisher Scientific Inc. (Waltham, MA, United States) and Merck Millipore (Billerica, MA, United States). The mutagenesis reaction contained 0.5 μl of Phusion High-Fidelity DNA Polymerase (2 U/μl) (Thermo Fisher Scientific, MA, Waltham, United States), 10 μl of 5 × Phusion HF-Puffer (Thermo Fisher Scientific, Waltham, MA, United States), 10 mM of each primer, 10 mM dNTPs (Thermo Fisher Scientific, MA, Waltham, United States), 14 μl of DMSO (Thermo Fisher Scientific, Waltham, MA, United States), 100 ng of plasmid DNA, and double-distilled water to a final volume of 50 μl. The probe was initially denatured at 98°C for 1 min, followed by 30 cycles, each containing denaturation at 98°C for 20 s, annealing at 60.6°C for 5 min, and elongation at 72°C for 8 min. The last step after finishing the PCR cycles was to hold 72°C for 10 min. Subsequently the parental template DNA of the generated PCR product was digested by adding 1 μl of *Dpn*1 endonuclease (10 U/μl) (Thermo Fisher Scientific, MA, Waltham, United States) followed by 1-h incubation at 37°C. The PCR product was analyzed by agarose gel electrophoresis and sequencing (GATC Biotech AG, Konstanz, Germany) to confirm the right mutation.

### Protein Purification

The purification of recombinant His-tagged protein from the cell culture media was performed by Ni-NTA affinity chromatography using Protino^TM^ Ni-NTA-Agarose (MACHEREY-NAGEL GmbH & Co. KG, Düren, Germany) in batch procedure. The purification was done according to the manufacturer’s protocol with minor changes. Briefly, the harvested cell culture medium was cleared from the remaining cells by centrifugation at 2,000 × *g*, 4°C for 5 min, and afterward cell debris was eliminated by centrifugation at 16,000 × *g*, 4°C for 45 min. The supernatant was added to the equilibrated Ni-NTA-Agarose beads, with NPI-10 buffer (50 mM NaH_2_PO_4_, 300 mM NaCl, and 10 mM imidazole, pH 8), and incubated for 1 h at 4°C. Protein-bound beads were washed in three washing steps, twice with NPI-30 (50 mM NaH_2_PO_4_, 300 mM NaCl, and 30 mM imidazole, pH 8) and the third time with an NPI-50 buffer (50 mM NaH_2_PO_4_, 300 mM NaCl, and 50 mM imidazole, pH 8). The recombinant protein was eluted in four steps, thrice with NPI-150 buffer 50 (50 mM NaH_2_PO_4_, 300 mM NaCl, and 150 mM imidazole, pH 8) and the last time with NPI-250 buffer (50 mM NaH_2_PO_4_, 300 mM NaCl, and 250 mM imidazole, pH 8). The collected eluent fractions were concentrated and finally the buffer was exchanged using Amicon Ultra-4, 50 K columns (Merck Millipore, Billerica, MA, United States).

### Purification of CD109 Containing EVs From Cell Culture Supernatant

To determine whether meprin β affects the amount and fragmentation of CD109, EVs were isolated for quantification purposes by using total exosome isolation reagent (Thermo Fisher Scientific, MA, Waltham, United States). The purification was performed according to the manufacturer’s protocol. The transfection was done as described above, medium was saved 40 h post transfection, centrifuged for 30 min at 2,000 × *g*, 4°C to eliminate remaining cells, and again at 10,000 × *g*, 4°C for 60 min to remove cell debris; 500 μl of isolation reagent was given to 1,000 μl of supernatant and incubated overnight at 4°C. The next day, the samples are centrifuged again for 60 min at 10,000 × *g*, 4°C, and the supernatant was discarded. EVs were resuspended in 50 μl of ice-cold DPBS and used for further analysis, such as Western blot.

For extracellular measurements in negative staining transmission electron microscopy (TEM) or for dynamic light scattering analysis (DLS), samples must be free from any matrix as it would be found in the abovementioned isolation kit. Therefore, EVs were isolated using ultracentrifugation as described previously (Arnold et al., 2020).

### TEM of EVs and Protein

Negative staining TEM was performed as described before (Arnold et al., 2014). The negative staining samples were prepared by using glow-discharged carbon-coated electron microscopy (EM) grids (Electron Microscopy **S**ciences, Hatfield, United States). The glow discharge was performed by using the Mini Sputter Coater System (Quorum Technologies, Lewes, United Kingdom), with a strong current of 25 mA for 30 s. The purified and previously buffer exchanged (100 mM Tris–HCl, pH 6.8) protein sample as well as the EV samples were added into the glow-discharged carbon-coated EM grid for 30 s, and access volume was removed using filter paper. The EM grid was stained twice with 1% aqueous uranyl acetate solution (Merck Millipore, Billerica, MA, United States), blotted again with filter paper, and air dried. The micrographs were collected by JEOL 1400 Plus TEM (JEOL Germany, Munich, Germany) operating at 100 kV with a nominal magnification of 50,000×.

### Single-Particle Reconstruction

For single-particle analysis, images were taken utilizing SerialEM (Mastronarde, 2005) and were then transferred to CisTEM (Grigorieff et al., 2017). After contrast transfer function (CTF) correction, single particles were selected automatically and class sum images were calculated. In the second round, particles from noisy classes were removed and 18.879 particles went into the final dataset. After class sum image formation, an initial model was calculated and then refined using the automated mode. The final reconstruction was filtered to 15 Å.

### Dynamic Light Scattering

For DLS analysis, EV samples, generated using ultracentrifugation, were adjusted to the same protein amount (measured as described above) and stored in DPBS. Samples were filled into precision cells made of Quartz SUPRASIL^®^ (Hellma GmbH & Co. KG, Müllheim, Germany) and measured with the Spectroscatter 201 (RiNA GmbH, Berlin, Germany). Each sample was prepared in three technical replicates and each was measured 10 times with a 90° scattering angle at 20°C.

### Terminal Isotopic Labeling of Substrates

Terminal Isotopic Labeling of Substrates (TAILS) analysis was performed as described by Becker-Pauly et al. (2011).

### Proteomics

#### Quantitative Proteomics of Cell Membranes and EVs

The provided proteins were fixated in gel for 15 min in a solution of 7% acetic acid and 40% methanol and subsequently stained for 15 min with a solution of 0.25% Coomassie Blue G-250 (Biozym Scientific GmbH, Hessisch Oldendorf, Germany), 7% acetic acid, and 45% ethanol. The gel was afterward washed several times with deionized water to remove excess dye. In-gel digestion was performed in principle as described previously (Shevchenko et al., 2006) and detailed as follows. Each gel lane was cut separately, minced, and transferred to an Eppendorf tube. Gel pieces were destained [50% ethanol in 25 mM NH_4_HCO_3_ (ABC)] for 15 min. The supernatant was removed, and the gel pieces were dehydrated by adding pure acetonitrile for 10 min with the tubes rotating in a rotator. The acetonitrile was removed first by pipetting and the samples were afterward dried to completion using a vacuum evaporator (Eppendorf AG, Hamburg, Germany). The dried samples were rehydrated, and disulfide bonds were reduced using reduction buffer [10 mM dithiotreitol (DTT) in 50 mM NH_4_HCO_3_, pH 8.0] for 1 h at 56°C. Buffer was removed by pipetting and cysteine residues of proteins were subsequently alkylated with 50 mM iodoacetamide (IAA) dissolved in 50 mM NH_4_HCO_3_, pH 8.0, for 45 min at room temperature (RT) in the dark. Samples were dehydrated again by adding pure acetonitrile and dried with vacuum evaporation. The vacuum-dried gel slices were incubated with 1 μg of trypsin per tube in 50 mM triethylammonium bicarbonate (TEAB) buffer, pH 8.0, at 37°C overnight. Digested peptides were extracted by adding twice 150 μl with 30% acetonitrile and once 150 μl of pure acetonitrile for 15 min at 25°C agitating at 1,400 rpm in a thermo shaker (Eppendorf AG, Hamburg, Germany). As the next step, we performed reductive dimethylation as described previously (Boersema et al., 2009) for each sample pair including a replicate with switched dimethyl labels. Equal amounts of peptides from all labeled samples were mixed. Purification and desalting were done using C18 material (M3 company) stage tips as previously described (Rappsilber et al., 2007). The eluted peptides were loaded on a column of 75 μm inner diameter (New Objective, FS360-75-8-N-5-C30) packed to 25 cm length with 1.9-μm Reprosil beads (Dr. Maisch HPLC GmbH, Ammerbuch, Germany) using the EasyLC1000 (Thermo Fisher Scientific, MA, Waltham, United States).

Peptides were separated on an EasyLC1000 HPLC (Thermo Fisher Scientific, MA, Waltham, United States) with the following reversed-phase chromatography gradient: 0–67 min 0–22% mixture B, 67–88 min 22–40% mixture B, and 89–92 min 40–95% mixture B (mixture A: 0.1% formic acid, mixture B: 80% acetonitrile containing 0.1% formic acid) and directly sprayed into a Q Exactive Plus mass spectrometer (Thermo Fisher Scientific, MA, Waltham, United States). The mass spectrometer was operating in positive scan mode with a full scan resolution of 70,000; AGC target 3 × 10^6^; max. IT = 20 ms; scan range 300–1,650 m/z with a top 10 MS/MS DDA method. Normalized collision energy was set to 25 and MS/MS scan mode was operated at a resolution of 17,000; AGC target 1 × 10^5^ and max IT of 120 ms. Database search was performed using MaxQuant (Cox and Mann, 2008). Version 1.5.2.8 with the *Homo sapiens* UniProt database. Trypsin/P was set as digestion mode allowing for two missed cleavages. Further settings were as follows: variable modification: Acetyl (Protein N-term), Oxidation (M); fixed modifications: Carbamidomethyl (C), FDR of 1% on peptide and protein level was applied.

For quantification, light label and heavy label were assigned on either lysine residues or N-terminal amino group with a maximum allowance for three labeled amino acids per peptide. Proteins with at least two peptides, one of them unique, were considered as identified. Proteins matching the reverse database or a common contamination list, as well as proteins containing only peptides with a modification, were removed. The quantification ratios from MaxQuant of the dimethyl labels were log2 transformed.

#### Identification of Meprin β Cleavage Sites—in-Gel N-Terminomics

The gel bands were washed and destained, reduced with DTT (10 mM), and alkylated with IAA (55 mM) using standard techniques, but in the presence of 50 mM HEPES (pH 7.5) rather than ABC. Protein N-terminus and lysine residues were reductively dimethylated using 40 mM formaldehyde (CH_2_O) and 20 mM sodium cyanoborohydride (NaBH_3_CN) for 3 h at 25°C for 3 h in HEPES buffer (pH 7.5). Excessive reagents were quenched in three washing steps: once with 50 mM ABC and two times with 30% ACN/50 mM ABC. Gel bands were dehydrated with ACN and vacuum centrifugation. Chymotrypsin (50 ng) was added, and the samples were digested overnight at 37°C in the presence of ABC. Peptides were extracted from the gel band using 1% formic acid (FA), 50% acetonitrile (ACN), 1% FA, and 100% ACN with the aid of sonication. Pooled supernatants were dried down by vacuum centrifugation and stored at −20°C and resuspended in 3% ACN and 0.1% trifluoroacetic acid (TFA) prior to analysis.

#### Quantitative Reductive Dimethylation of Meprin β-Cleaved CD109

Briefly, 1.5 μg of CD109 transfected with active (mep) and inactive meprin β (E153A) was made up to 30 μl in a denaturing buffer 100 mM TEAB and 1% SDS and inactivated by the addition of Tris(carboxyehtyl)phosphine (TCEP, 5 mM) at 95°C for 10 min. Samples were reduced for another 30 min and then alkylated with IAA (12.5 mM) for 30 min at RT and then quenched with DTT. Samples were precipitated onto a mixture of SpeedBeads, Magnetic Carboxylate particles (GE Healthcare, Chicago, IL, United States), and then labeled with light (CH_2_O, meprin β) and heavy (CD_2_O, meprin β E153A) formaldehyde in the presence of NaCNBH_3_, as described previously (Weng et al., 2019). After labeling, samples were quenched, precipitated on fresh beads, and then digested with 100 ng of trypsin overnight at 37°C in 100 mM TEAB buffer. Samples were combined at this stage and dried down and resuspended prior to analysis, as described above.

#### LC/MS Measurements

Samples were injected on a Dionex Ultimate 3000 nano-UHPLC coupled to either a Q Exactive HF mass spectrometer or Q Exactive Plus (Thermo Fisher Scientific Inc., Waltham, MA, United States). The samples were washed on a trap column (Acclaim Pepmap 100 C-18, 5 mm × 300 μm, 5 μm, 100 Å, Dionex) for 4 min with 3% ACN/0.1% TFA at a flow rate of 30 μl/min prior to peptide separation using an Acclaim PepMap 100 C-18 analytical column (50 cm × 75 μm, 2 μm, 100 Å, Dionex). A flow rate of 300 nl/min using eluent A (0.05% FA) and eluent B (80% ACN/0.04% FA) was used for gradient separation (5–40% B). Spray voltage applied on a metal-coated PicoTip emitter (10 μm tip size, New Objective, Woburn, MA, United States) was 1.6 kV, with a source temperature of 250°C. For Q Exactive Plus, measurements were acquired at a resolution of 70,000 at m/z 200. The 10 most intense precursors with charge states ≥ 2 + were subjected to fragmentation with HCD with NCE of 27%; isolation width of 3 m/z; resolution, 17,500 at m/z 200. For measurements on the Q Exactive HF Full scan, MS spectra were acquired between 350 and 1,400 m/z at a resolution of 120,000 at m/z 200. The 10 most intense precursors with charge states greater than 2 + were selected with an isolation window of 1.4 m/z and fragmented by HCD with normalized collision energies of 27 at a resolution of 30,000. Lock mass (445.120025) and dynamic exclusion (30 s) were enabled on both machines.

#### Database Search

The MS raw files were processed by Proteome Discoverer 2.2 (Thermo Fisher Scientific Inc., Waltham, MA, United States, version 2.2.0.388) and MS/MS spectra were searched using the Sequest HT algorithm against common contaminants and the canonical and reviewed human database. An MS1 tolerance of 10 ppm and an MS2 tolerance of 0.02 Da was implemented. Carbamidomethylation (57.02146 Da) on cysteine residues and dimethylation on lysine residues (28.031 Da) were set as fixed modifications while oxidation of methionine (15.995 Da) residues and dimethylation on the peptide N-terminus were set as dynamic modifications. For in-gel N-terminomics, samples were searched with semi-chymotrypsin specificity with three missed cleavages allowed, while for quantitative reductive dimethylation, the sample was searched with semi Arg-C specificity with two missed cleavages allowed. For quantitative reductive dimethylation, two sequest nodes were used to search the data: one node with “light” dimethylation (+28.031 Da) set as dynamic modification on peptide N-termini, and as a fixed modification on lysine residues, while the other implemented “heavy” dimethylation (+32.056 Da) on the aforementioned lysine residues and peptide N-termini. High-confidence peptides were set to a 1% FDR, while medium-confidence peptides were allowed a 5% FDR. For in-gel dimethylation, N-terminal peptides were only considered if they were identified with at least two PSMs.

The mass spectrometry (MS) proteomics data have been deposited to the ProteomeXchange Consortium^1^ via the PRIDE partner repository (Perez-Riverol et al., 2019) with the dataset identifier PXD023727.

### Bioinformatics and Statistics

#### Sequence Retrieval of CD109 Protein

The sequence for the structural study of CD109 protein was retrieved from UniProt sequence database^2^ (Apweiler et al., 2010). The UniProt ID for CD109 protein sequence was Q6YHK3.

#### Model Building and Evaluation of in Silico Homology Models for CD109 Protein

The modeling of the three-dimensional structure of CD109 protein was performed by a homology modeling program Swiss-Model^3^ (Bordoli et al., 2009). The CD109 protein amino acid sequence (UniProt ID: Q6YHK3) in the range of 22–1,420 was used as a target for the search of modeling templates. The quality of the generated three-dimensional homology models was evaluated using QMEAN *Z*-score estimate^4^ (Benkert et al., 2009) and Ramachandran plot analysis^5^ (Prisant et al., 2003).

#### Statistics

If not differently specified, the results are given as mean ± standard error of the mean (SEM). To determine if two groups of data were significantly different, unpaired non-parametric Mann–Whitney *U* rank test was used. Due to small collectives testing for a normal distribution could not be performed. *p* < 0.05 was considered as statistically significant. Statistical analysis was performed by using GraphPad Prism 6.0 (GraphPad Software, San Diego, CA, United States).

## Results

### Cleavage of CD109 by Meprin β

CD109 was identified during a TAILS approach using the keratinocyte-derived HaCaT cell line (Becker-Pauly et al., 2011). The cleavage sites identified were found in the bait region (E666/E667 and N668/E669) and between L1283/D1284, which fit the cleavage site preference of meprin β, i.e., with a negatively charged residue in P1’ position (Becker-Pauly et al., 2011). The cleavage site is located in the bait region of CD109 (Figure 1A) and is in close proximity to a previously reported cleavage site for BMP-1 (Delolme et al., 2015). As TAILS detects cleavage events triggered by the protease of interested, we were keen to understand if cleavage of CD109 by meprin β could be quantified on a global protein level. For this, we treated cells with recombinant meprin β (Becker et al., 2003) and compared protein levels of the cellular membrane fraction of these cells with cells not treated with meprin β. Quantitative proteomics revealed a reduced CD109 cell surface amount among the most abundant ∼1,600 proteins upon incubation with meprin β (Figure 1B). Thus, we conclude that CD109 is an important substrate that encounters a global cell surface downregulation after cleavage by meprin β. To determine if CD109 could be cleavage *in vitro*, recombinant CD109 (#4385-CD-050, R&D Systems, Minneapolis, MN, United States) was incubated with recombinant meprin β, the closely related meprin α (Becker-Pauly et al., 2007) and BMP-1, a protease known to process CD109 (Moali et al., 2005). Major cleavage of CD109 was detected for meprin β in Coomassie-stained SDS-gel analysis, resulting in two major protein fragments, one running at 120 kDa and one at 70 kDa (Figure 1C). For meprin α and BMP-1, only minor cleavage of CD109 was detected, as majority of full-length CD109 was still observed following meprin α and BMP-1 incubation (Figure 1C). In-gel reductive dimethylation followed by chymotrypsin digestion and MS analysis was performed on the two CD109 bands generated by meprin β to identify their N-termini. The 120-kDa fragment corresponds to the N-terminus and the 70-kDa fragment to the C-terminus of CD109 (Figure 1D and Supplementary Figure 1A). Moreover, for the 70-kDa C-terminal fragment of CD109, several dimethylated N-termini in the bait region were identified, namely, M665/E666, E667/N668, N668/E669, and H676/D677 that fit the cleavage preference for meprin β (Figure 1E and Supplementary Figure 1B). To test cleavage of membrane-bound CD109 by membrane-bound meprin β, HEK293T cells were co-transfected with CD109 and increasing amounts of active wild-type (wt) meprin β or an inactive variant of meprin β where glutamate 153 was exchanged to an alanine (E153A; Wichert et al., 2019). To identify the N-terminal cleavage fragment, an N-terminal HA-tagged CD109 was used for co-expression experiments. For this, CD109 and meprin β (wt or E153A) were transiently transfected, and in cell lysates, a CD109 fragment was detected in Western blot analysis in samples co-expressing CD109 and active meprin β running at around 120 kDa (Figure 1F). Upon precipitation of the supernatant using ConA (Concanavalin A) conjugated beads and Western blot analysis using an anti-HA-tag antibody, N-terminal fragments were detected at around 120 and 50 kDa and cleavage can clearly be attributed to meprin β activity as both fragments are missing for the samples derived from the inactive (E153A) meprin β variant (Figure 1F). Probing the same membrane with a CD109-specific antibody revealed an additional cleavage fragment, running at ∼70 kDa, that must come from the core of the protein, as the N-terminus (HA-tag) is missing (Figure 1F). The full-length version of CD109 was also detected in the supernatant, which was reported before (Hagikura et al., 2010) and can be due to cleavage of the GPI anchor or release of CD109 carrying EVs. To determine the cleavage site of the meprin β-dependent N-terminal fragment and for further experiments, two different HEK293T cell lines were generated that stably express differently tagged CD109 (Supplementary Figures 1C,D). One cell line expresses CD109 N-terminally tagged with an HA-tag (N-HA-CD109 HEK293T; Figure 1G and Supplementary Figures 1C–D), and one cell line was generated with an N-terminally His-tagged CD109 (N-His-CD109 HEK293T) for purification of the N-terminal fragment via Ni-NTA precipitation (Figure 1H and Supplementary Figure 1F). Both cell lines showed good resistance against the selection antibiotic hygromycin B, demonstrating the successful selection for stably expressing cells. After transfection of the N-HA-CD109 HEK293T cells with meprin β and the inactive E153A meprin β, cleavage fragments in the supernatant were detected at 50 and 70 kDa (Figure 1G). Lack of the 120-kDa fragment after re-probing the membrane with HA-tag or CD109 antibodies (Figure 1G and Supplementary Figure 1E) might indicate that this larger fragment is transient and will be reduced into the two smaller ones under steady-state protein expression. To determine if membrane-bound meprin β also cleaves within the bait region, N-His-CD109 HEK293T cells were transfected with the two meprin β variants, and after Ni-NTA purification of cleavage fragments from the supernatant and SDS-PAGE followed by Coomassie staining, clear signals for meprin β-dependent cleavage fragments were detected (Figure 1H and Supplementary Figure 1F). To identify the cleavage sites, we performed quantitative proteomics using light (CH_2_O) and heavy (CD_2_O) labeling for meprin β and the E153A variant, respectively, and identified a cleavage site between 676H and 677D that was unique for CD109 transfected with meprin β (Supplementary Figures 1G,H). This confirms that *in vivo* membrane-bound meprin β also cleaves CD109 in the bait region (Supplementary Figure 1H). To summarize, using different proteomics approaches, we identified several cleavage sites within the bait region all fitting to the cleavage preference of meprin β, but only cleavage site H676/D677 was found in the recombinant *in vitro* and in the cell culture experiments.

### Homology Modeling and Single-Particle Reconstruction of CD109

**FIGURE 2:**
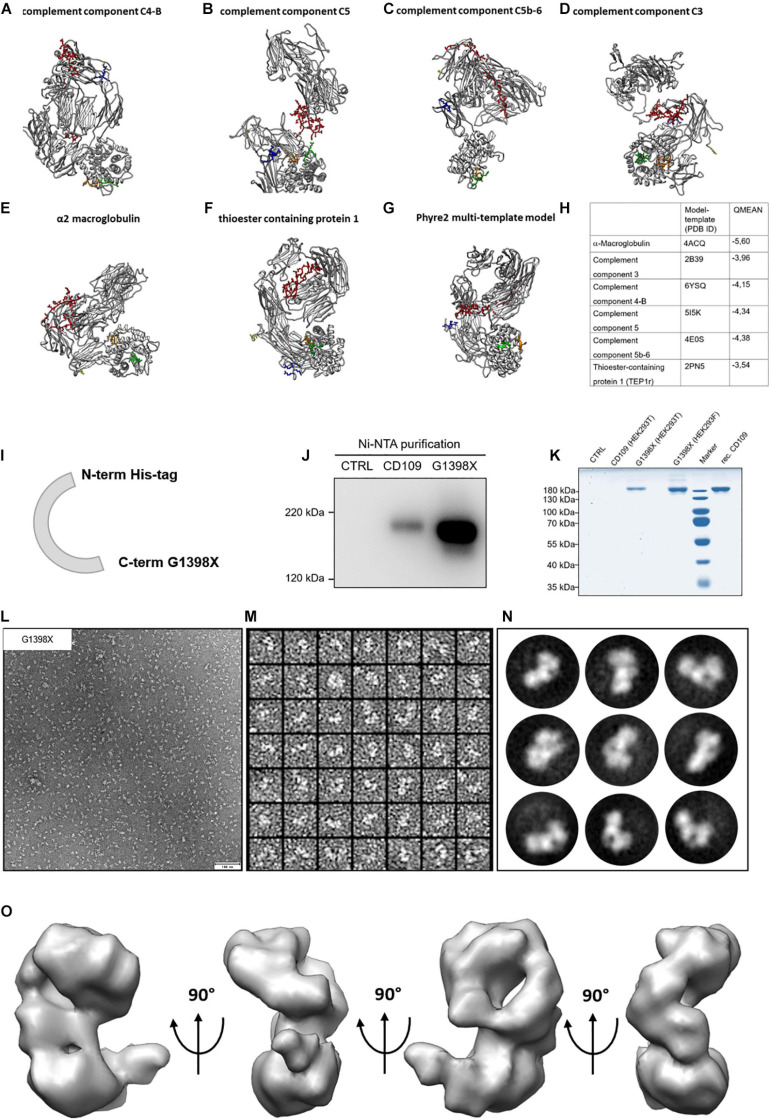
Homology models for CD109 and single-particle reconstruction of CD109 from negative stain images. **(A–F)** *In silico* homology models for CD109 acquired with Swiss-Model ExPASy. **(G)** The multi-template approach utilizing Phyre2 resulted in yet another structure model for CD109. **(H)** The used templates and descriptions with QMEAN *Z* scores. The colored regions show specific functional areas of the CD109 protein (yellow: C-terminus; red: bait region; orange: thioester region; green: reactive thioester motif; blue: furin cleavage motif). **(I)** Schematic illustration of the CD109 (G1398X) construct without GPI anchor and N-terminal His-tag. Comparison of purified cell supernatants of full-length CD109 and G1398X variant to control and purchased recombinant protein from R&D Systems shown in Western blot analysis **(J)** and Coomassie gel **(K)** after transient transfection in HEK293T and Freestyle HEK293F cells compared to recombinant (rec.) CD109. **(L)** Representative EM micrograph of negative stained purified CD109 variant G1398X without GPI anchor recorded with SerialEM software. **(M)** Representative image of extracted single particles of CD109 protein variant G1398X. The software *cis*TEM was used for processing the EM data and imaging of 2D class averages of negative stained CD109 variant G1398X **(N)**. Final 3D model of CD109 with a resolution of 15 Å vertically rotated in a 90° angle **(O)**.

The cleavage sites identified for meprin β that are located within the bait region are found in the middle of the primary sequence (around amino acid 667 from 1445). Cleavage of CD109 by meprin β at this position seems not to be a shedding event, where ectodomains of membrane-bound proteins are released by meprin β from the cell surface (Arnold et al., 2017; Bedau et al., 2017a). To structurally localize the cleavage sites for meprin β and also BMP-1 (Delolme et al., 2015) within the bait region, we used homology modeling (SwissModel and Phyre2) and searched automatically for templates to gain insights into the CD109 structure. The highest sequence identity was found with α2 macroglobulin (∼29%) and phylogenetic analysis also groups CD109 closest to the α2 macroglobulin family (Lin et al., 2002). The other templates identified were components of the complement system, a group of proteins that also contain a thioester reactive group (Gadjeva et al., 1998). Additionally, the thioester binding protein 1 (TEP1r) from *Drosophila* [ortholog to CD109, (Minata et al., 2019)] was identified as a suitable template with ∼23% sequence identity. All models calculated with SwissModel (Figures 2A–F) display different folds, and the bait region (Figures 2A–G, red) is in different positions of the protein. The multi-template model generated by Phyre2 (Figure 2G) showed yet another fold, however, with a similar conformation as the one determined for the TEP1r template. Comparison of the resulting structures according to the QMEAN score (Figure 2H) revealed the best value for the model derived from the TEP1r template (pdb#: 2PN5). Although correlation of model and template and Ramachandran blot analysis was conducted (Supplementary Figure 2A), none of the structures appeared superior over the other ones. Thus, we decided that unbiased structural information is required and generated a soluble version of CD109 (sCD109) devoid of the GPI anchor (ending at amino acid G1398) and equipped with an N-terminal 10 × His-tag for Ni-NTA purification using a site-directed mutagenesis approach. This resulted in a single-nucleotide exchange (G4192T), changing the codon at this position into a stop codon (Supplementary Figure 2B). After testing for normal transport of this sCD109 G1398X variant (Figure 2I) through the cellular secretion pathway (Supplementary Figures 2C,D), it was also detected in the supernatant and could be enriched after Ni-NTA purification, as detected by Western blot analysis (Figure 2J). Purity was determined by Coomassie-stained SDS-PAGE and appeared suitable (>95%) for structure determination (Figure 2K). For superior protein expression, we also changed into HEK293F suspension cells, as larger protein amounts could be purified from them. For structure determination, freshly purified sCD109 was applied onto a negatively glow-discharged carbon-coated TEM grid and was negatively stained using uranyl-acetate. At a nominal resolution of 4.58 Å/pixel, images were taken (Figure 2L) and transferred to *cis*TEM for single-particle processing. After CTF correction, particles were selected (Figure 2M; about 18.000 particles in final dataset) and class sum images were calculated (Figure 2N). The resulting model was refined using the automated mode and filtered to a final resolution of 15 Å (Figure 2O).

### Structure Interpretation of CD109

**FIGURE 3:**
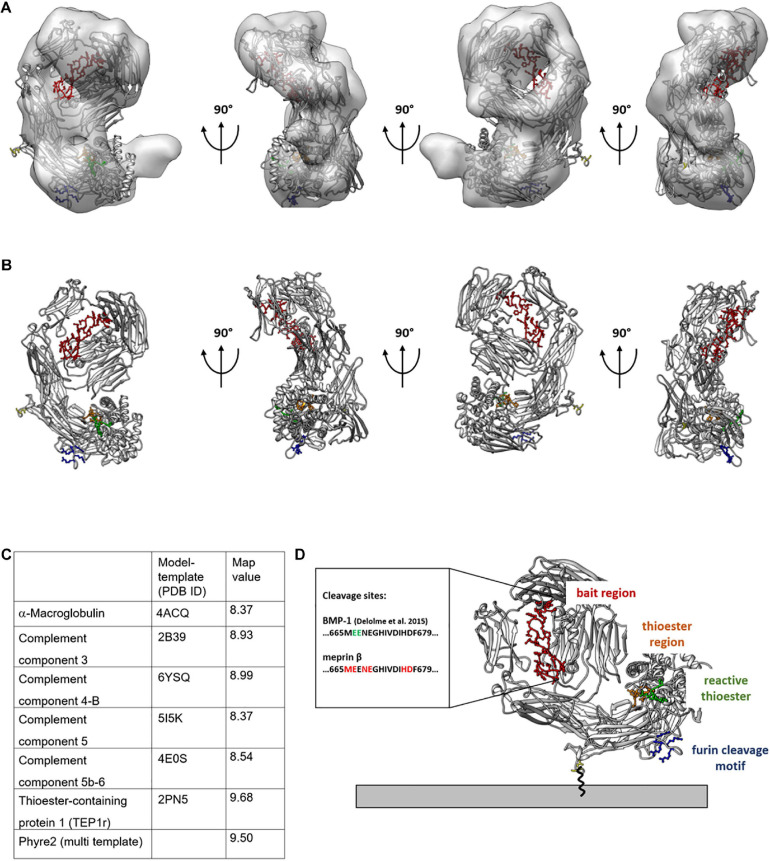
Molecular model of TEP1 fits best into 3D reconstruction of CD109. Thioester-containing protein 1 (TEP1r, PDB ID: 2PN5) matches best with the negative stain transmission electron microscopy (TEM)-generated 3D model. 2PN5 homology model is outlined within the 3D model **(A)** or by itself **(B)** rotated in 90° angles with colored functional regions; legend shown in panel **(D)**. For quality reasons, map values were calculated and displayed in tabular view in panel **(C)**. C-terminus expressed in yellow gives an idea of the structural orientation of CD109 on the cell surface **(D)** Connected to the GPI anchor. The bait region is shown in red with possible cleavage sites given for meprin β and BMP-1; the thioester region is in orange, and its structural proximity to the reactive thioester-defining hexapeptide motif is in green; the furin cleavage site oriented toward the cell membrane is in blue.

To determine the molecular model that best fits the calculated 3D reconstruction, we docked all calculated homology models into the 3D density and calculated the average map value (UCSF Chimera) (Figure 3A and Supplementary Figure 3). This value is representative for the number of atoms within the density and the higher a value, the more atoms are within the 3D reconstruction. For the model calculated from the TEP1r protein (pdb#: 2PN5), a good overall correlation of the molecular model and the calculated 3D reconstruction was determined (Figure 3A). However, a small part of the 3D reconstruction was not accounted for by the molecular model. As the furin cleavage site is in close proximity to the unfilled mass (Figures 3A,B, blue residues), cleavage of CD109 by furin might also induce a small molecular rearrangement, resulting in a different conformation in this region of the protein. For all other molecular models calculated from the abovementioned templates, inferior average map values were calculated (Figure 3C). Although there is still uncertainty in higher-resolution orientation, we can now propose a molecular model for CD109 based on homology modeling and single-particle reconstruction (Figure 3D). This model puts the C-terminal end toward the membrane attached to a GPI anchor. The furin cleavage motif with the four positively charged arginine residues (RRRR) is in close proximity to the membrane (Figure 3D, blue residues), and these residues could also stabilize CD109 on the membrane by interacting with the negatively charged cell surface. The bait region (Figure 3D, red residues) forms a large loop that is accessible at the side of CD109. Within this loop, cleavage sites for BMP-1 (Delolme et al., 2015) and meprin β can be found. The thioester region (Figure 3D, orange residues) and the reactive thioester-defining hexapeptide motif (Figure 3D, green residues) reside within the α-helical thioester domain.

### Proteolytic Cleavage Controls CD109 Levels on EVs

**FIGURE 4:**
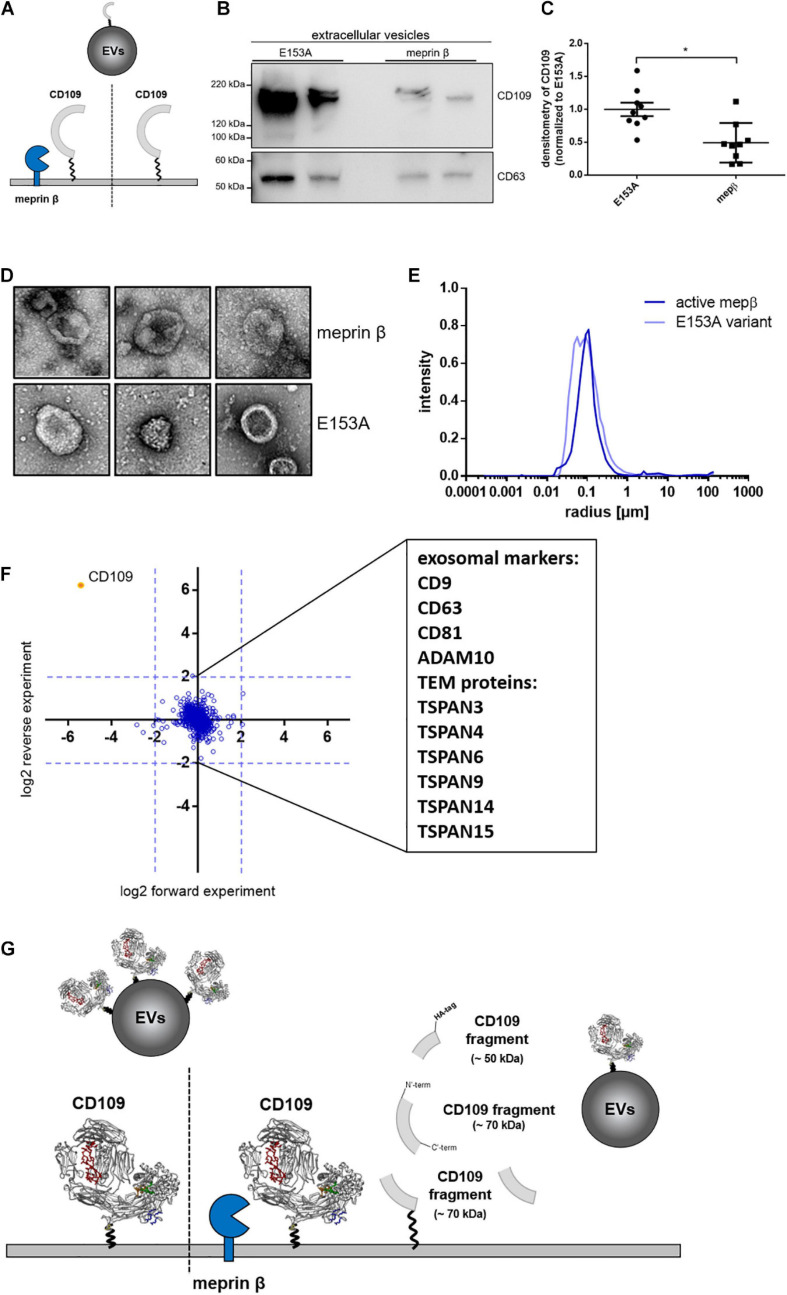
Amount of CD109 on extracellular vesicles reduced after meprin β cleavage. To determine differences of extracellular vesicle (EV) composition between active meprin β and its inactive variant E153A after transient transfection of N-term HA-tagged CD109 stable HEK293T cells [schematic view in panel **(A)**], an exosome purification kit was used to extract EVs from cell supernatant. Representative Western blot (*n* = 9) is shown in panel **(B)** and quantification to exosome marker CD63 (as internal standard control) and afterward normalized to inactive meprin β control **(C)**; statistical analysis was performed using Mann–Whitney test, *p* < 0.05 was considered statistically significant, *p < 0.01. In panels **(D–F)**, extracellular vesicles were extracted via ultracentrifugation and analyzed in negative stain transmission electron microscopy for size and shape **(D)** as well as dynamic light scattering analyses; results show no differences in mean radius distribution **(E)**. In the proteomics approach, many exosome markers and tetraspanin-enriched microdomain (TEM) proteins could be detected, but no distinct protein could be detected up- or downregulated besides CD109 **(F)**. In summary, **(G)** gives a schematic overview of meprin β influencing CD109 not only on the cell surface but also on extracellular vesicle transport.

It was shown before that CD109 can be sorted to exosomes (Sakakura et al., 2016) and increasing levels of full-length CD109 circulating in patients suffering from non-small cell lung carcinoma (Zhang et al., 2014). We were interested whether proteolysis controls the amount of CD109 secreted on EVs (Figure 4A). For this, we purified EVs from stably CD109-expressing cells transfected with active or inactive (E153A) meprin β. We found that EVs derived from cells expressing active meprin β show reduced CD109 levels (Figure 4B), which was also significant when first analyzed against the exosome marker CD63 (as internal standard control) and afterward normalized to E153A (Figure 4C). To gain a better insight into the EVs, we analyzed them by TEM (Figure 4D) and DLS (Figure 4E). Both methods showed no differences between the two EV populations. To determine whether CD109 forms a protein complex prior to sorting onto EVs, we compared EVs that express CD109 and EVs from control cells by quantitative MS (Figure 4F). As expected, we found a strong upregulation for CD109, but no other protein was additionally co-sorted to EVs in a CD109-dependent manner. We also identified many exosome markers such as CD9, CD63, CD81, and ADAM10 (Figure 4F) and also identified many tetraspanins (TSPAN) enriched in tetraspanin-enriched microdomains (Figure 4F, TEM proteins) such as TSPAN3, 4, 6, 9, 14, and 15. The identification of these TSPANs is not surprising, as exosomes derive from tetraspanin-enriched microdomains at the cell surface (Andreu and Yáñez-Mó, 2014).

## Discussion

The cell surface protein CD109 can be proteolytically processed at the cell surface by BMP-1 within the bait region (Delolme et al., 2015). We also found cleavage within this region for meprin β (and to minor extend by meprin α) and moreover discovered that an additional cleavage event takes place within the N-terminal part, resulting in ∼50-kDa and ∼70-kDa fragments present in the supernatant. Structural assessment of CD109 revealed a closer relationship to the *Drosophila* CD109 ortholog TEP1 and not to α2-macroglobulin, as suggested from sequence alignments (Lin et al., 2002). A previously suggested domain distribution for CD109 based on the structure of a C3 component showed a similar localization of the reactive thioester and the thioester binding domain but does not resolve the bait region and could therefore not be further interpreted (Janssen et al., 2005). The exact structural arrangement with a defined localization of all secondary structures, however, will only be possible from a better resolved experimentally determined structure (single particle or crystallization). The overall orientation of CD109 on the cell surface as proposed here fits the cleavage preference of membrane-bound meprin β as this protease is not only a classical sheddase that liberates substrate ectodomains from the cell membrane but can also process cleavage sites located further away from the membrane as shown for the IL-6 receptor (Arnold et al., 2017) and CD99 (Bedau et al., 2017a,b). The orientation of the bait region also fits the currently accepted model of membrane-bound meprin β (Arolas et al., 2012). Whether cleavage induces a conformational change, as observed for α2 macroglobulin (Marrero et al., 2012), will have to be determined in future studies.

Soluble CD109 has been observed in patient’s serum suffering from non-small cell lung carcinoma and was associated with a poor prognosis (Taki et al., 2020). CD109 was also found to be upregulated in glioblastoma after radiation induced damage and stabilized the cancer stem cell niche (Minata et al., 2019). Here, CD109 was suggested to act upstream of the Yap/Taz signaling pathway (Minata et al., 2019). In a *Drosophila* glioma model, it was also shown that TEP1 (*Drosophila* ortholog of human CD109) regulates the *Drosophila* Yki pathway (ortholog to Yap/Taz) (Gangwani et al., 2020). This functional consensus of TEP1 and CD109 might also favor a structural similarity between both proteins. It was previously demonstrated that CD109 can be sorted onto EVs (Sakakura et al., 2016; Yamamoto et al., 2019), and here we can show that proteolytic activity at the cell surface reduces this CD109 secretion mechanism. It was shown previously for the IL-6 receptor (Arnold et al., 2020) and the Prion protein (Linsenmeier et al., 2018) that proteolytic activity controls the amount of membrane proteins sorted onto EVs. The vesicular morphology was, however, unchanged when comparing CD109 containing or non-containing vesicles, unlike for the release of PRDX4, where larger vesicles were detected (Lipinski et al., 2019). As no other cell surface protein was detected to be co-sorted to EVs together with CD109, we suggest that CD109 might have oncogenic properties itself. Further, as a GPI-anchored protein, CD109 should reside within lipid rafts (Hagiwara et al., 2008). However, CD109 sorted onto EVs most likely originates from tetraspanin-enriched microdomains, as determined by MS experiments. Thus, CD109 might not reach its natural interaction partners on the cell surface, prior to sorting to EVs.

Taken together, we provide a comprehensive analysis of CD109 fates at the cell surface. We demonstrate how proteolytic processing at the cell surface reduces the amount of full-length CD109 sorted to EVs. Proteolysis results in the release of fragmented CD109 into the supernatant, and using single-particle analysis and homology modeling, we could also provide the first experimentally solved structure of CD109, allowing us to deduce its orientation on the cell membrane and the identified cleavage sites.

## Data Availability Statement

The datasets presented in this study can be found in online repositories. The name of the repository and accession number can be found below: PRoteomics IDEntification Database (PRIDE), https://www.ebi.ac.uk/pride/, PXD023727.

## Author Contributions

WL and PA: conceptualization. WL, SB, TK, EP, FP, PS, and PA: formal analysis and investigation. WL and PA: writing—original draft preparation. All authors: writing—review and editing. CB-P, FZ, and PA: funding acquisition. CB-P, RL, and FZ: resources. PA: supervision.

## Conflict of Interest

The authors declare that the research was conducted in the absence of any commercial or financial relationships that could be construed as a potential conflict of interest.

## Footnotes

1 http://proteomecentral.proteomexchange.org

2 https://www.uniprot.org

3 https://swissmodel.expasy.org

4 https://swissmodel.expasy.org/qmean/

5 http://mordred.bioc.cam.ac.uk/∼rapper/rampage.php

## Supplementary Material

Supplementary Figure 1 CD109 cleavage by meprin β – proteomics data and CD109-stable HEK293T cells. Recombinant CD109 digested with meprin β, meprin α, and BMP-1 in a Coomassie stained SDS-PAGE gel **(A)**. Sequence coverage of the two bands of CD109 following meprin β incubation and in-gel N-terminal dimethylation and chymotryptic digestion. The 120 kDa band (band 5) belongs to the N-terminus while the 70 kDa band (band 6) belongs to the protein’s C-terminus. The difference in protein weight may be explained by the high rate of N-glycosylations (shown in red), which reside more on the N-terminal half of CD109. N-terminal dimethylated peptides that were identified in CD109 control and in band 6. Several false positives or minor degradation products were observed in the control band. **(B)** Dimethylated N-terminal peptides with at least two Peptide Spectra Matches (PSMs) observed for CD109 (band 1) and upon addition of meprin β (band 6). N-terminal peptides that were observed with a high number of PSMs and not observed in the control sample were considered as potential meprin β cleavage sites, e.g., M.EENEGHIVDIHDF, N.EGHIVDIHDFSLGSSPHVRKHFPETW and H.DFSLGSSPHVRKHFPETW with 4, 34 and 6 PSMs respectively. **(C)** Western blot showing cell lysates of differently tagged CD109 stable polyclonal cells after 2 weeks of hygromycin B treatment and in panel **(D)** monoclonal CD109 stable HEK293T cells each compared to normal transient transfection with CD109 HA-tag or pcDNA as control incubated and detected with CD109 antibody. **(E)** Western blot of cell lysates corresponding to supernatant samples shown in Figure 1G. **(F)** Additional to section with Ni-NTA purification of Coomassie gel already shown in Figure 1H, the whole gel shows comparison between native medium, followed by the purification steps extracellular vesicles (EVs) via ultracentrifugation, ConA precipitation, and His-tag purification (via Ni-NTA) compared to 1 μg purchased recombinant (rec.) CD109. The marked signal at ∼70 kDa was analyzed using mass spectrometry. Upon in-solution quantitative reductive dimethylation we were able to confirm one of the potential cleavage sites that was identified in-gel. Extracted ion chromatographs of N-terminally dimethylated peptide H.DFSLGSSPHVR is a result of cleavage by meprin β at 676H.677D **(G)**, therefore it is clearly more abundant in the meprin β sample **(H)**.

Supplementary Figure 2 Quality assessment of homology models and result of PCR based site directed mutagenesis and G1398X construct verification. Overview of generated homology models and quality assessment via QMEAN local quality score and global QMEAN Z score **(A)**. The models and associated sequences are colored according to the local quality (blue: high quality, red: low quality). Additionally, local QMEAN estimates are shown for the generated 3D-structures. The stereo-chemically quality of modeled structures was evaluated via general Ramachandran-Plot analysis. **(B)** Identification of the expression vector for CD109 without GPI-anchor after PCR based site-directed mutagenesis via sequencing, followed by fractionation experiment scheme **(C)** for the extraction of separated cell components as well as cell supernatant fractions after transient transfection of HEK293T cells with expression plasmids for CD109 variants and empty vector as negative control. **(D)** The verification of CD109 variants localization in the individual fractions was performed via western blot analysis (representative blot is shown out of a series of *n* = 3).

Supplementary Figure 3 Comparison of other homology models fitted into 3D reconstruction of CD109. The other possible homology models were fitted into the 3D reconstruction and rotated in a 90° angle as described in Figure 3.
