# Supplementary figures and images for: Cell Surface Processing of CD109 by Meprin β Leads to the Release of Soluble Fragments and Reduced Expression on Extracellular Vesicles
Source: Front Cell Dev Biol. 2021 Mar 2;9:622390. doi: 10.3389/fcell.2021.622390 (PMC7960916; doi:10.3389/fcell.2021.622390)

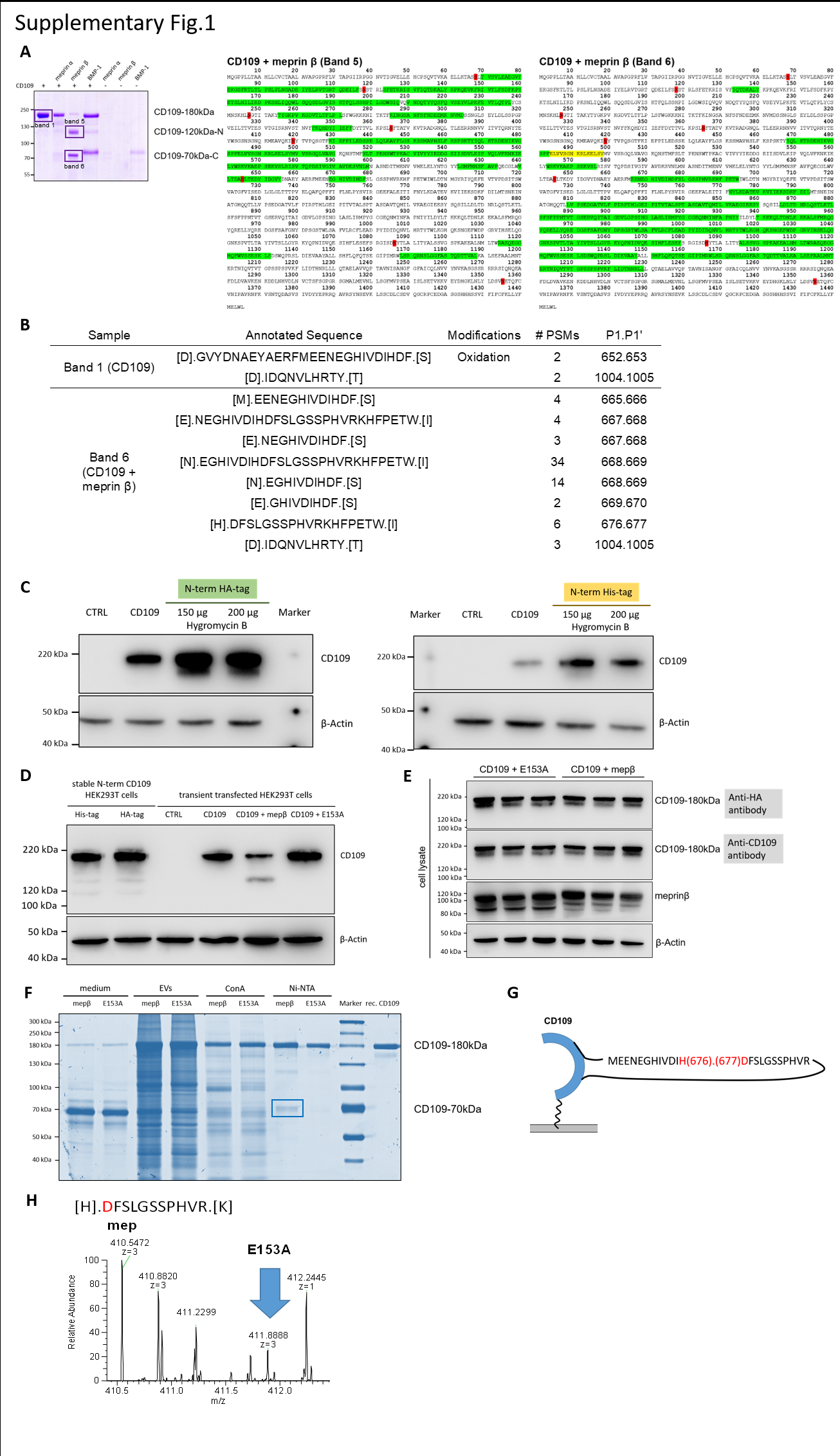

Supplement: Supplementary Figure 1 — CD109 cleavage by meprin β – proteomics data and CD109-stable HEK293T cells. Recombinant CD109 digested with meprin β, meprin α, and BMP-1 in a Coomassie stained SDS-PAGE gel (A). Sequence coverage of the two bands of CD109 following meprin β incubation and in-gel N-terminal dimethylation and chymotryptic digestion. The 120 kDa band (band 5) belongs to the N-terminus while the 70 kDa band (band 6) belongs to the protein’s C-terminus. The difference in protein weight may be explained by the high rate of N-glycosylations (shown in red), which reside more on the N-terminal half of CD109. N-terminal dimethylated peptides that were identified in CD109 control and in band 6. Several false positives or minor degradation products were observed in the control band. (B) Dimethylated N-terminal peptides with at least two Peptide Spectra Matches (PSMs) observed for CD109 (band 1) and upon addition of meprin β (band 6). N-terminal peptides that were observed with a high number of PSMs and not observed in the control sample were considered as potential meprin β cleavage sites, e.g., M.EENEGHIVDIHDF, N.EGHIVDIHDFSLGSSPHVRKHFPETW and H.DFSLGSSPHVRKHFPETW with 4, 34 and 6 PSMs respectively. (C) Western blot showing cell lysates of differently tagged CD109 stable polyclonal cells after 2 weeks of hygromycin B treatment and in panel (D) monoclonal CD109 stable HEK293T cells each compared to normal transient transfection with CD109 HA-tag or pcDNA as control incubated and detected with CD109 antibody. (E) Western blot of cell lysates corresponding to supernatant samples shown in Figure 1G. (F) Additional to section with Ni-NTA purification of Coomassie gel already shown in Figure 1H, the whole gel shows comparison between native medium, followed by the purification steps extracellular vesicles (EVs) via ultracentrifugation, ConA precipitation, and His-tag purification (via Ni-NTA) compared to 1 μg purchased recombinant (rec.) CD109. The marked signal at ∼70 kDa was analyzed using mass [file Image_1.tif]

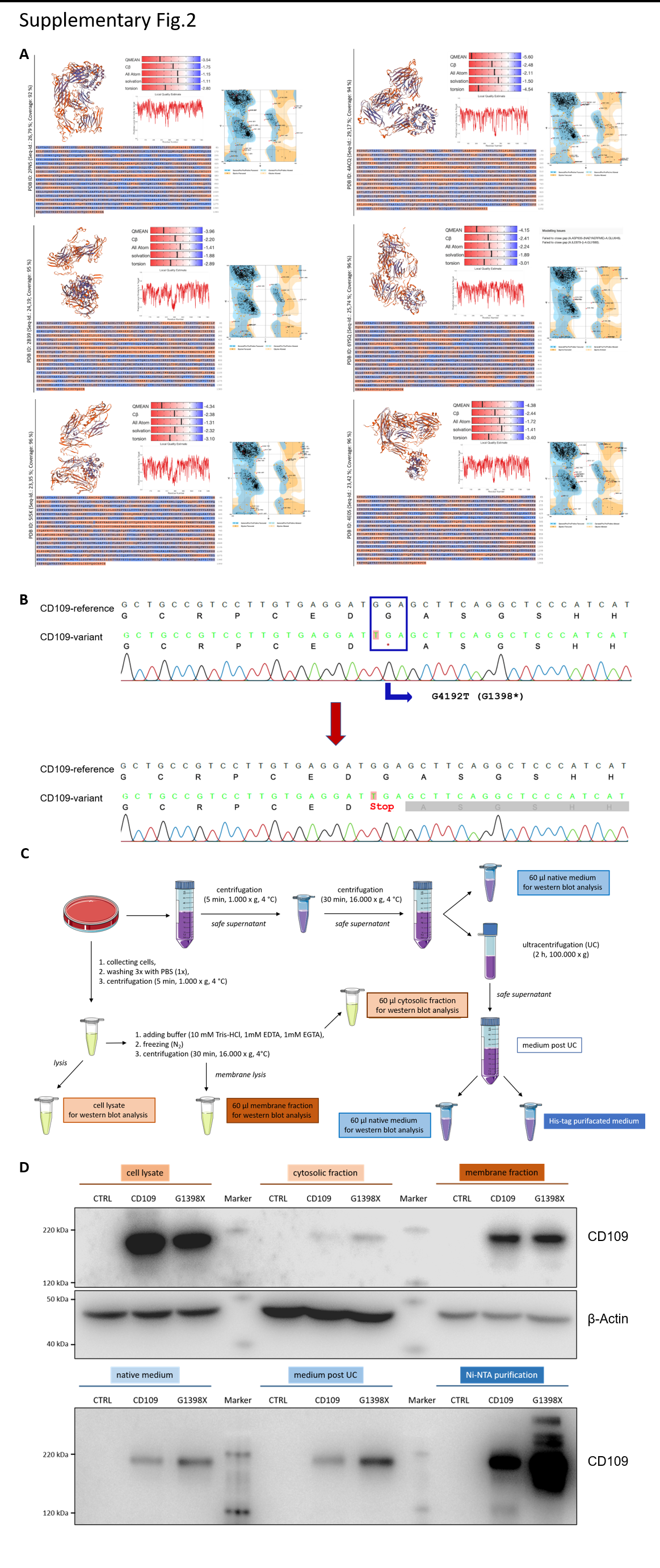

Supplement: Supplementary Figure 2 — Quality assessment of homology models and result of PCR based site directed mutagenesis and G1398X construct verification. Overview of generated homology models and quality assessment via QMEAN local quality score and global QMEAN Z score (A). The models and associated sequences are colored according to the local quality (blue: high quality, red: low quality). Additionally, local QMEAN estimates are shown for the generated 3D-structures. The stereo-chemically quality of modeled structures was evaluated via general Ramachandran-Plot analysis. (B) Identification of the expression vector for CD109 without GPI-anchor after PCR based site-directed mutagenesis via sequencing, followed by fractionation experiment scheme (C) for the extraction of separated cell components as well as cell supernatant fractions after transient transfection of HEK293T cells with expression plasmids for CD109 variants and empty vector as negative control. (D) The verification of CD109 variants localization in the individual fractions was performed via western blot analysis (representative blot is shown out of a series of n = 3). [file Image_2.tif]

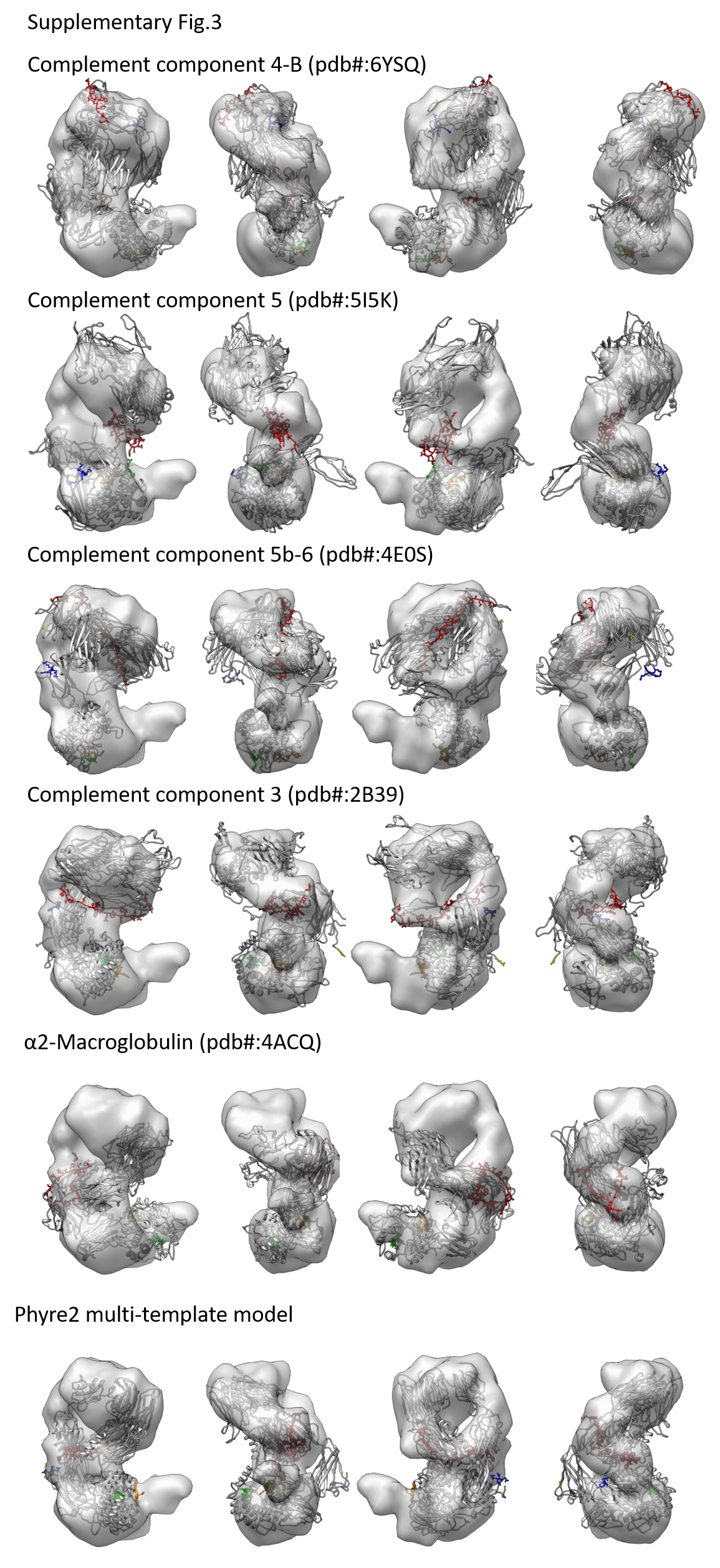

Supplement: Supplementary Figure 3 — Comparison of other homology models fitted into 3D reconstruction of CD109. The other possible homology models were fitted into the 3D reconstruction and rotated in a 90° angle as described in Figure 3. [file Image_3.PNG]
